# Effect of Functional Oligosaccharides and Ordinary Dietary Fiber on Intestinal Microbiota Diversity

**DOI:** 10.3389/fmicb.2017.01750

**Published:** 2017-09-20

**Authors:** Weiwei Cheng, Jing Lu, Boxing Li, Weishi Lin, Zheng Zhang, Xiao Wei, Chengming Sun, Mingguo Chi, Wei Bi, Bingjun Yang, Aimin Jiang, Jing Yuan

**Affiliations:** ^1^College of Food Science, South China Agricultural University Guangzhou, China; ^2^Institute of Disease Control and Prevention, Academy of Military Medical Sciences Beijing, China; ^3^Department of Laboratory, Yuhuangding Hospital Yantai, China

**Keywords:** functional oligosaccharides, intestinal microbiota, diversity, fecal metabolic profiling, microecosystem

## Abstract

Functional oligosaccharides, known as prebiotics, and ordinary dietary fiber have important roles in modulating the structure of intestinal microbiota. To investigate their effects on the intestinal microecosystem, three kinds of diets containing different prebiotics were used to feed mice for 3 weeks, as follows: GI (galacto-oligosaccharides and inulin), PF (polydextrose and insoluble dietary fiber from bran), and a GI/PF mixture (GI and PF, 1:1), 16S rRNA gene sequencing and metabolic analysis of mice feces were then conducted. Compared to the control group, the different prebiotics diets had varying effects on the structure and diversity of intestinal microbiota. GI and PF supplementation led to significant changes in intestinal microbiota, including an increase of *Bacteroides* and a decrease of *Alloprevotella* in the GI-fed, but those changes were opposite in PF fed group. Intriguing, in the GI/PF mixture-fed group, intestinal microbiota had the similar structure as the control groups, and flora diversity was upregulated. Fecal metabolic profiling showed that the diversity of intestinal microbiota was helpful in maintaining the stability of fecal metabolites. Our results showed that a single type of oligosaccharides or dietary fiber caused the reduction of bacteria species, and selectively promoted the growth of *Bacteroides* or *Alloprevotella* bacteria, resulting in an increase in diamine oxidase (DAO) and/or trimethylamine N-oxide (TMAO) values which was detrimental to health. However, the flora diversity was improved and the DAO values was significantly decreased when the addition of nutritionally balanced GI/PF mixture. Thus, we suggested that maintaining microbiota diversity and the abundance of dominant bacteria in the intestine is extremely important for the health, and that the addition of a combination of oligosaccharides and dietary fiber helps maintain the health of the intestinal microecosystem.

## Introduction

The human gastrointestinal tract contains about 500–1,500 different species of gut microorganisms (Lozupone et al., 2012). Gram-negative and anaerobic bacteria account for the majority of organisms, and are present in numbers approximately 100–1,000 times those of aerobic bacteria. These “guest” flora are dominated by four main phyla of bacteria: Firmicutes, Bacteroidetes, Actinobacteria, and Proteobacteria (Gill et al., 2006). They are important for maintaining our homeostasis. The intestinal microbiota is an important bridge between diet and human health, and can be thought of as a “microbial organ” (O’Hara and Shanahan, 2006) that helps ferment undigested food, face aggressor microorganisms, maximize the energy that can be extracted from nutrients, and produce essential nutrients/vitamins that we are not equipped to produce (Gill et al., 2006). The number and structure of the intestinal microflora is affected by various factors, such as intestinal pH, peristalsis, body nutrition status, age, and health status (Roberfroid et al., 2010). Maintaining the balance within the microbial community is essential for human health, and perturbation of this balance can induce diseases, including obesity, metabolic dysfunction, and immune system disorders (Cani et al., 2008; Vijay-Kumar et al., 2010; Frank et al., 2011; Wang et al., 2015). The composition of the intestinal microflora is determined by the individual genotype (Kovacs et al., 2011) and other environmental factors (De Filippo et al., 2010; Muegge et al., 2011; Bolnick et al., 2014), with diet being particularly important.

Probiotics and prebiotics can improve type 2 diabetes and cardiovascular disease by improving the intestinal microbiota, which leads to insulin signal stimulation and lower cholesterol levels. Adding prebiotics to the diet can adjust the intestinal microecological environment, and thereby promote the proliferation of probiotics in the intestine. Although the use of probiotics to improve the composition of the gut microbiota is known to be beneficial, there are still many deficiencies in our knowledge, including how these probiotic species colonize the gut, whether they are destroyed during passing through the upper gastrointestinal tract, their residence time in the intestine, and the probability of probiotic product being produced. Prebiotics can be used to aid the stable proliferation of beneficial bacteria, allowing the gut microflora to maintain a good micro-ecological balance (Brownawell et al., 2012; Guarner et al., 2012).

Although the mechanism of the selective stimulation of *Bifidobacterium bifidum* growth by prebiotics remains unknown, many studies also show that oligosaccharides in the intestine can be fermented and utilized by various bacteria, including *Streptococcus* (Hartemink et al., 1995), *Escherichia* (Hartemink et al., 1997), and *Clostridium* (Van Laere et al., 2000) species. Therefore, the United Nations Food and Agriculture Organization (FAO) Prebiotics special meeting (Pineiro et al., 2008) defined prebiotics as inanimate food ingredients with the ability to regulate the intestinal microbial flora and benefit the host’s health. This definition confirmed that the improvement of the intestinal micro-ecological environment should not just be attributed to stimulation of a specific type of microorganism, such as bifidobacteria, but should involve the intestinal microbiota as a whole. Prebiotics include galacto-oligosaccharides (GOS), fructo-oligosaccharides, and inulin, amongst others.

Dietary fiber, a polysaccharide-based macromolecular substance, is a necessary nutrient that is broken down in the intestine via bacterial fermentation. Although it is not easily digested or absorbed by the human body, its volume and ability to absorb water can promote intestinal peristalsis, thereby preventing constipation. Dietary fiber is used as a carbon source by intestinal bacteria, providing nutrition and energy to the host. However, because dietary fiber assists the growth of both beneficial and harmful bacteria, and has no selectivity, it cannot be called a prebiotic. GOS are functional oligosaccharides with natural properties, and are abundant in human breast milk. While inulin is found in chicory fiber (Van et al., 1995), not all dietary fibers contain prebiotics (Lee et al., 2015). In the current study, these general dietary fibers are referred to as ordinary dietary fiber. Most previous studies have focused on the effects of oligosaccharides on given gut microbes, with few studies examining the effects of oligosaccharides and ordinary dietary fiber on the gut flora as a whole. Therefore, more information is needed to identify the “ideal” diet for a healthy gut microbiome (Dey, 2017) and to determine whether the addition of prebiotics or ordinary dietary fiber to the diet could improve the health of the human body. To identify prebiotic- or dietary fiber-related factors that contribute to the maintenance of a healthy gut microbiota, we performed a dietary intervention study in which we compared three different combinations of feed additives: GI (GOS and inulin), PF (polydextrose and insoluble dietary fiber), and a GI/PF mixture (GI and PF, 1:1). The effects of these three combinations on the gut microecology were also investigated.

## Materials and Methods

### Animals and Sample Collection

Eight-week-old male BALB/c mice were obtained from the Laboratory Animal Center, Academy of Military Medical Sciences (Beijing, China) and maintained in an IVC rodent caging system. The mice were housed in a temperature- and humidity-controlled (18–24°C, 50–60%, respectively) room under a strict 12 h light cycle (lights on at 8:00 h and off at 20:00 h). Each group was fed with the indicated diet *ad libitum*, with free access to water. All of the protocols used in this study were approved by the Ethics Committee of the Academy of Military Medical Sciences (Beijing, China) and the procedures were carried out in accordance with the European Community guidelines for the care and use of experimental animals.

**FIGURE 1:**
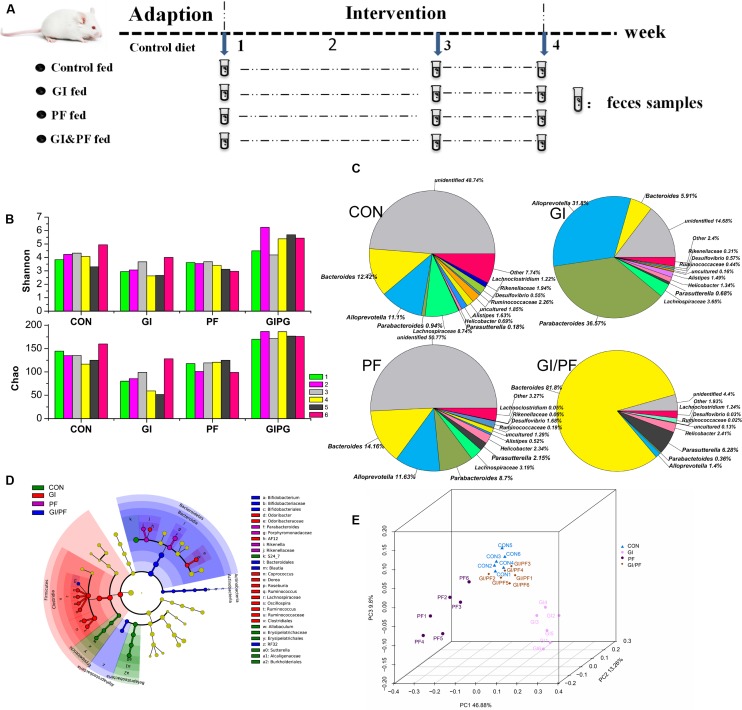
Effects of the three treatments on the composition of fecal microbiota. **(A)** Experimental design. **(B)** A plot of the Shannon–Wienner diversity and Chao index at 4-week point. **(C)** Relative abundance of the main genera at 4-week point. **(D)** Analysis of taxonomic abundances using LEfSe indicates that multiple taxa are differentially enriched in the feces of four groups. **(E)** A PCoA analysis of the UniFrac distance in the feces of four groups. GI, galacto-oligosaccharides + inulin group; PF, polydextrose + insoluble dietary fiber group; GIPF, GI and PF supplementation at a 1:1 ratio group. “0” indicates the first week. “1” indicates the third week. “2” indicates the fourth week.

The twenty-four 10-week-old male BALB/c mice were randomly divided into four groups (*n* = 6): control group, GI group, PF group, and GI/PF group. The average body weight was 25 g. The mice were adapted to the new environment for 1 week, followed by a 3-week intervention period. To prepare the supplemented feed, diet pellets (Laboratory Animal Center, Academy of Military Medical Sciences) were broken up and amended with functional oligosaccharides and/or ordinary dietary fiber in different proportions, and then the pellets were reformed. As shown in **Figure 1A**, the control group was fed the control diet and the other three groups were fed separate diets supplemented with GI (7.5% GOS and 7.5% inulin), PF (7.5% polydextrose and 7.5% insoluble dietary fiber), or GI/PF (7.5% GI and 7.5% PF) during the intervention period (**Table 1**). At the time points indicated in **Figure 1A**, mice were transferred to separate sterilized cages, and feces were collected in individual sterile EP tubes stored on ice, which were then taken to the laboratory within 2 h of collection for further study. Blood was collected from the orbital vein, and serum was obtained by centrifugation at 1,000 × *g* for 20 min at room temperature. After 4 weeks, mice were fasted for 12 h prior to sacrifice, and the small intestine was removed and stored in 10% formalin solution until processing into wax blocks. The small intestine was stained with hematoxylin and eosin (HE) to examine the villus structure. The colon was opened longitudinally, and the content was collected by scraping and stored at -80°C for the determination of pH value.

**Table 1:** Composition of experimental diets.

	CON(g/kg)	GI(g/kg)	PF(g/kg)	GI/PF(g/kg)
Protein	180	180	180	180
Fat	40	40	40	40
Starch	650	500	500	500
Moisture	130	130	130	130
GOS	–	75	–	37.5
Inulin	–	75	–	37.5
Polydextrose	–	–	75	37.5
Insoluble dietary fiber (bran)	–	–	75	37.5


*CON, control group; GI, galacto-oligosaccharides + inulin group; PF, polydextrose + insoluble dietary fiber group; GI/PF, GI and PF supplementation at a 1:1 ratio group*.

### DNA Extraction, PCR Amplification, and Illumina MiSeq Sequencing

Microbial DNA was extracted from fecal samples using an E.Z.N.A. fecal DNA Kit (Omega Bio-tek, Norcross, GA, United States) according to the manufacturer’s protocol. The V4–V5 region of the bacterial 16S ribosomal RNA gene was amplified by PCR (95°C for 2 min, followed by 25 cycles of 95°C for 30 s, 55°C for 30 s, and 72°C for 30 s, with a final extension at 72°C for 5 min) using primers 515F (5′-barcode-GTGCCAGCMGCCGCGG-3′) and 907R (5′-CCGTCAATTCMTTTRAGTTT-3′), where the barcode was an 8-bp sequence unique to each sample. Illumina adapters: P5 AATGATACGGCGACCACCGAGATCTACAC[i5]TCGTCGGCAGCGTCAGATGTGTATAAGAGACAG;P7CAAGCAGAAGACGGCATACGAGAT[i7]GTCTCGTGGGCTCGGAGATGTGTATAAGAGACAG. PCR were performed in triplicate 20-μL mixtures containing 4 μL of 5× FastPfu Buffer, 2 μL of 2.5 mM dNTPs, 0.8 μL of each primer (5 μM), 0.4 μL of FastPfu DNA Polymerase, and 10 ng of template DNA. Amplicons were extracted from 2% agarose gels and purified using an AxyPrep DNA Gel Extraction Kit (Axygen Biosciences, Union City, CA, United States) according to the manufacturer’s instructions, and then quantified using the QuantiFluor-ST system (Promega, United States). Purified amplicons were pooled in equimolar concentrations and paired-end sequenced (2 × 250) on an Illumina MiSeq platform according to standard protocols. The raw reads were deposited into the NCBI Sequence Read Archive (SRA) database (accession number: SRP113656).

### Processing of Sequencing Data

Raw fastq files were demultiplexed and then quality-filtered using QIIME (version 1.9.1) with the following criteria: (i) the 300-bp reads were truncated at any site receiving an average quality score <20 over a 50-bp sliding window, discarding the truncated reads that were shorter than 50 bp; (ii) exact barcode matching: reads containing a 2-bp mismatch during primer matching or reads containing ambiguous characters were removed; (iii) only sequences that overlapped by >10 bp were assembled according to their overlap sequence. Reads that could not be assembled were discarded.

Operational taxonomic units were clustered with a 97% similarity cutoff using UPARSE^1^ (version 7.1), and chimeric sequences were identified and removed using UCHIME. The taxonomy of each 16S rRNA gene sequence was analyzed by RDP Classifier^2^ against the silva (SSU115) 16S rRNA database using a confidence threshold of 70% (Amato et al., 2013).

### Fecal Metabolic Profiling

Aliquots (100 mg) of each fecal sample were weighed out, and a 1-mL volume of methanol:water:chloroform (3:1:1) was added to each sample. Following homogenization, ultrasonic extraction was carried out for 15 min on ice. After standing for 4 h, the supernatant was collected by centrifugation at 10,000 × *g*. A 100-μL volume of the supernatant was mixed with 20 μL of 0.2 mg/mL ribose alcohol as an internal standard, thoroughly mixed, and then dried at 45°C with N_2_. A 40-μL volume of 20 mg/mL methoxyamine solution was added to the dried extract, thoroughly mixed, and then incubated at 30°C for 90 min at 130 rpm. After the reaction was complete, 40 μL of Bis(trimethylsilyl)trifluoroacetamide (containing 1% trimethyl chlorosilane) were added, and the samples were mixed thoroughly. After being incubated for 30 min at 37°C, the samples were incubated for a further 2 h at room temperature, and then at 4°C for detection. Gas chromatography-mass spectrometry (GC-MS) analysis was then performed under the following conditions: Agilent 7890 series LECO Pegasus 4D time-of-flight (TOF)/MS detector. Gerstel MPS injection system. Column: DB-5MS 30 m × 250 μm × 0.25 mm. Program temperature: 70°C, hold 1 min, increase 5°C/min to 280°C, hold 10 min. Carrier gas: He. Flow rate: 1 mL/min. Injection volume: 1 μL. Split ratio 1:2. MS conditions: electron ionization source. Mass scanning range: 50–800 Da, scanning speed 10/s. Inlet, transmission line, and ion source temperature: 250, 250, and 220°C, respectively.

Chromatography of the compounds was performed, and chromatographic peak alignment, deconvolution, and peak retrieval (carrying National Institute of Standards and Technology) and other mass spectrometry database and related standards for identification) was conducted using the Chroma TOF 4.50 workstation. Ribose alcohol was used as the internal standard, with a signal to noise ratio of >100.

### Determination of Colon Content pH and Serum Concentrations of SCFA, DAO, and TMAO

Aliquots (100 mg) of colon content were weighed out and mixed with deionized water at 15 mL/g (Depenbusch et al., 2008). The samples were centrifuged at 13,000 × *g* for 2 min, and then the pH of the supernatant was measured using a pH meter. Serum concentrations of short-chain fatty acids (SCFA), diamine oxidase (DAO), and trimethylamine N-oxide (TMAO) were determined using mouse SCFA ELISA, DAO ELISA, and TMAO ELISA kits (Mlbio, Shanghai, China), respectively.

### Analysis of Villus Height and Crypt Depth in the Small Intestine

The small intestine samples were fixed in 10% formalin and then processed in wax blocks. The small intestine were stained with HE for the histological studies. Villus length and crypt depth in the intestinal tissue were analyzed using Image pro-plus 6.0 (Media Cybernetics, United States).

### Statistical Analysis

The differences among groups were analyzed using a one-way ANOVA with Tukey’s honestly significant difference *post hoc* test. The multivariate analyses were performed using SIMCA14.1 software package (V14.1, MKS Data Analytics Solutions, Umea, Sweden) to generate a OPLS-DA model with all the variables, and quality controls were predicted into this model. The variable importance in the projection (VIP) values exceeding 1.0 were selected as changed metabolites (Supplementary Table S1, *p* < 0.05). The analyses were performed using SPSS software (version 18.0.; SPSS Inc., Chicago, IL, United States).

## Results

### Ecological Diversity of Fecal Microbiota

Bacteria belonging to seven different phyla were identified in the mouse fecal samples, and Firmicutes and Bacteroidetes were the predominant phyla. At the genus level, *Bacteroides* and *Alloprevotella* were the two most abundant Bacteroidetes genera during the GI intervention period. However, during this period, there was a significant increase in the number of *Bacteroides* bacteria, but a significant decrease in the overall number of *Alloprevotella* (**Figure 1C**, *p* < 0.01). During the PF intervention period, a significant decrease in *Bacteroides* but an increase in *Alloprevotella* and *Parabacteroides* species was observed (*p* < 0.05). Compared with observations after 1 week of adaption, significant increases in *Bacteroides*, *Alloprevotella*, and *Parabacteroides* species were observed in the fecal samples of GI/PF-fed mice after 3 weeks of GI/PF-fed supplementation (*p* < 0.05). Changes in microbial communities were investigated using the Shannon–Wienner diversity and Chao index. GI and PF significantly lowered the Shannon–Wienner diversity and Chao index when compared to CON, whereas GIPF increased it (**Figure 1B**). Therefore, supplementation with functional oligosaccharides and ordinary dietary fiber resulted in an overall decrease in the diversity of the intestinal microbiota, while mixed supplementation (i.e., GI/PF) increased and then maintained the diversity (**Figure 1C**). We noted by LEfSe that cladogram showed those taxonomies that are significantly enriched in four groups (**Figure 1D**). This is consistent with the above analysis. Significant increases in the prevalence of *Bifidobacterium* species were also observed in the GI and GI/PF treatment groups, although their overall abundance remained low (increased from 0 to 0.04%). By PCoA analysis, we found that the four groups showed a distinct separation and that the spatial location of control-fed and GIPF-fed groups was similar, indicating the flora structure was similar in both groups (**Figure 1E**).

### Fecal Metabolic Profiling of the Three Treatment Groups

**FIGURE 2:**
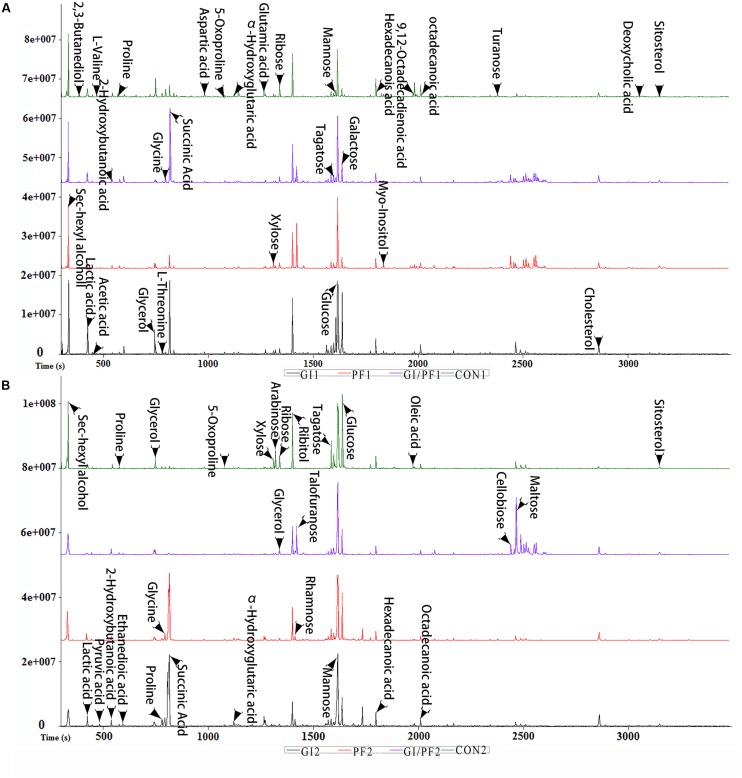
Typical base peak intensity chromatograms for each experimental group. **(A)** Supplementation intervention for 2 weeks. **(B)** Supplementation intervention for 3 weeks.

The fecal metabolic profiles of all experimental mice were obtained by GC-MS analysis. The typical base peak intensity chromatograms for each experimental group are shown in **Figure 2**. Based on comparisons of the ratio of the peak area between the experimental groups and the control group, supplementation with functional oligosaccharides and/or ordinary dietary fiber led to significant biochemical changes in the fecal microbiota. In all, 16 metabolites were identified as potential dietary-induced biomarkers (VIP > 1, *p* < 0.05, Supplementary Table S1). Variations in the biomarkers are described in **Table 2**. For example, increases in succinic acid, α-hydroxyglutaric acid, glycine, myo-inositol, and cholesterol were observed after 3 weeks of dietary intervention in GI-fed and PF-fed groups, compared with the control group. Glyoxylate and dicarboxylate metabolism along with inositol phosphate metabolism appeared to be significantly involved in these changes. Mannose, galactose, and 5-oxoproline were decreased after 3 weeks of supplementation in PF-fed groups, compared with the control group. Interestingly, decreases in proline, α-hydroxyglutaric acid, and glycine were observed in the GI/PF treatment group compared with the control, while these three metabolites were increased in the GI and PF treatment groups. And five amino acids (L-isoleucine, L-leucine, norvaline, L-tyrosine, L-phenylalanine) are significantly reduced with the rational combination of components (Supplementary Table S2).

**Table 2:** Trends in levels of potential fecal biomarkers in experimental mice.

Metabolites	GI1/CON	PF1/CON	GIPF1/CON	GI2/CON	PF2/CON	GIPF2/CON	Pathways
Succinic acid	7.01	16.34	9.50	16.79	25.49	1.11	Glyoxylate and dicarboxylate metabolism
Proline	4.53	0.45	8.22	20.37	13.81	0.43	Arginine and proline metabolism
9,12-Octadecadienoic acid	0.24	0.44	0.24	12.68	9.11	10.90	Linoleic acid metabolism
Myo-inositol	10.34	7.35	8.60	12.35	8.54	8.47	Inositol phosphate metabolism
Cholesterol	1.90	1.57	1.54	7.06	4.52	1.70	Steroid hormone biosynthesis
α-Hydroxyglutaric acid	1.63	0.60	1.31	5.86	2.68	0.57	Glyoxylate and dicarboxylate metabolism
Glycine	1.26	0.25	0.83	4.89	3.52	0.31	Primary bile acid biosynthesis
2-Hydroxybutanoic acid	2.74	1.93	1.03	2.78	1.58	1.92	Propanoate metabolism
Glucose	8.21	1.79	2.71	1.92	1.81	4.02	Carbohydrate digestion and absorption
Pyruvic acid	–	–	–	1.16	0.91	1.47	Pyruvate metabolism
Lactic acid	2.20	0.87	1.30	2.05	1.13	1.21	Butanoate metabolism
Octadecanoic acid	0.72	0.59	0.64	1.54	0.74	1.08	Fatty acid biosynthesis
L-Aspartic acid	0.25	0.21	0.19	2.81	0.68	1.21	Alanine, aspartate, and glutamate metabolism
5-Oxoproline	0.47	0.45	0.37	2.91	0.02	0.54	Glutathione metabolism
Mannose	1.44	1.32	1.25	1.10	0.45	1.01	Amino sugar and nucleotide sugar metabolism
Galactose	7.70	0.00	22.66	0.34	0.18	0.14	Amino sugar and nucleotide sugar metabolism/galactose metabolism


*GI1/CON, the ratio of the values from GI and Control animals after 2 weeks of supplementation, indicating fold change in the level of metabolites; PF1/CON, the ratio of the values from PF and Control animals after 2 weeks of supplementation; GIPF1/CON, the ratio of the values from GIPF and Control animals after 2 weeks of supplementation; GI2/CON, the ratio of the values from GI and Control animals after 3 weeks of supplementation, indicating fold change in the level of metabolites; PF2/CON, the ratio of the values from PF and Control animals after 3 weeks of supplementation; GIPF2/CON, the ratio of the values from GIPF and Control animals after 3 weeks of supplementation*.

### Correlation between the Gut Microbiota and Fecal Metabolic Phenotype

**FIGURE 3:**
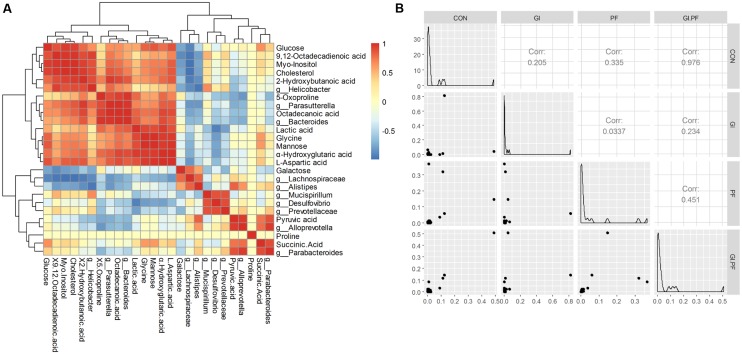
The Spearman correlation analysis between the abundance of fecal metabolites **(A)** and fecal microbiota genera composition **(B)** among the groups.

Functional oligosaccharide and/or ordinary dietary fiber supplementation of the mouse diet changed the structure/composition of the gut microbiota and substantially altered the fecal metabolic phenotype (**Figure 3A**). We found *Bacteroides*, enhanced in the fecal microbiota community in GI-fed group, was positively correlated with proline (*R* = 0.71), α-hydroxyglutaric acid (*R* = 0.88), lactic acid (*R* = 0.83), 2-hydroxybutanoic acid (*R* = 0.85). *Alloprevotella* and *Parabacteroides* contained an increased abundance in PF-fed group, was positively correlated with succinic Acid (*R* = 0.77, *R* = 0.89). *Parasutterella*, had a decreased abundance in PF-fed group, was positively correlated with 9,12-octadecadienoic acid (*R* = 0.73), 5-oxoproline (*R* = 0.88), octadecanoic acid (*R* = 0.98), L-aspartic acid (*R* = 0.84). *Mucispirillum* were all positively correlated with proline (*R* > 0.97) and 9,12-octadecadienoic acid (*R* > 0.51). Mannose was all positively correlated with *Bacteroides* (*R* = 0.71) and *Parasutterella* (*R* = 0.67). Galactose as all positively correlated with Lachnospiraceae (*R* = 0.79) and *Alistipes* (*R* = 0.56). As shown in **Figure 3B**, the overall correlation analysis showed that the control group was positively correlated with the GI/PF-fed group (*R* = 0.98, **Figure 3B**).

### pH and SCFA Concentrations of Colon Contents

**FIGURE 4:**
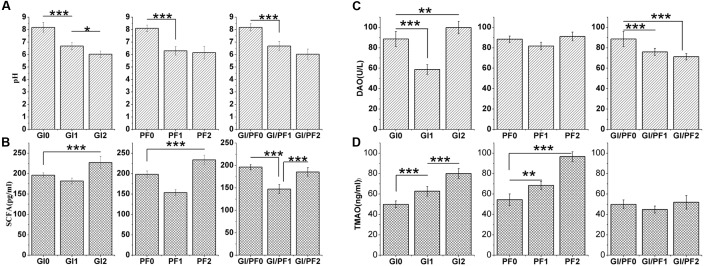
**(A)** Effects of the three treatments on the pH of the colon contents. **(B)** Effects of the three treatments on the short-chain fatty acid levels in the colon contents. **(C)** Effects of the three treatments on diamine oxidase concentrations in serum. **(D)** Effect of the three treatments on trimethylamine N-oxide concentrations in serum. The data represent the mean ± SD. ^∗^*p* < 0.05, ^∗∗^*p* < 0.01, ^∗∗∗^*p* < 0.005.

The pH levels of the colon contents of GI-fed mice were significantly lower (*p* < 0.005) after 2 and 3 weeks of supplementation (GI1 and GI2 time points, respectively) than that observed after 1 week of adaption (GI0 time point; **Figure 4A**). The same trend was observed for the PF and GI/PF groups. There was also a significant decrease (*p* < 0.05) in pH between weeks 3 and 4 in the GI treatment group. Thus, oligosaccharides, ordinary dietary fiber, and a combination of the two appeared to decrease the pH of the colon contents.

The GI2 mice displayed SCFA concentrations that were significantly higher (*p* < 0.005) than those observed at the GI0 and GI1 time points (**Figure 4B**). While the SCFA levels increased in all groups between the third and fourth weeks (*p* < 0.005), there were differences in the overall trends of the three groups. In addition, compared with levels after 1 week adaption, the mice fed the GI/PF diet for 3 weeks had no significant change in SCFA levels. In general, the SCFA content in the mouse colon increased with the decrease in pH, except in the GI/PF mice.

### DAO and TMAO Concentrations in Serum

The serum of GI-fed mice displayed significantly lower (*p* < 0.005) concentrations of DAO after 2 weeks of supplementation than after 1 week adaption, while at the GI2 time point, mice had significantly higher (*p* < 0.01) DAO values than at the GI0 time point (**Figure 4C**). The serum levels of DAO and TMAO in the PF group did not change significantly across the three time periods, while the DAO levels of the GI/PF group gradually decreased (*p* < 0.001) over the 4-week period (**Figure 4C**). In contrast, the TMAO levels of the GI mice increased over the 4 weeks (*p* < 0.005, **Figure 4D**). Compared with the 1-week time point, the TMAO concentrations in the serum of PF-fed mice were significantly increased by 4 weeks (*p* < 0.005). There was no significant difference in TMAO concentration between GI/PF2 and GI/PF0 mice, while the TMAO concentration in the serum of GI/PF1 mice was significantly decreased. Therefore, prolonged use of oligosaccharides or dietary fiber appeared to increase the TMAO content, while to a certain extent, the combination of GI and PF appeared to help reduce the serum concentration of TMAO.

### Effect of Three Treatments on Small Intestinal Villus Height and Fossae Depth

**FIGURE 5:**
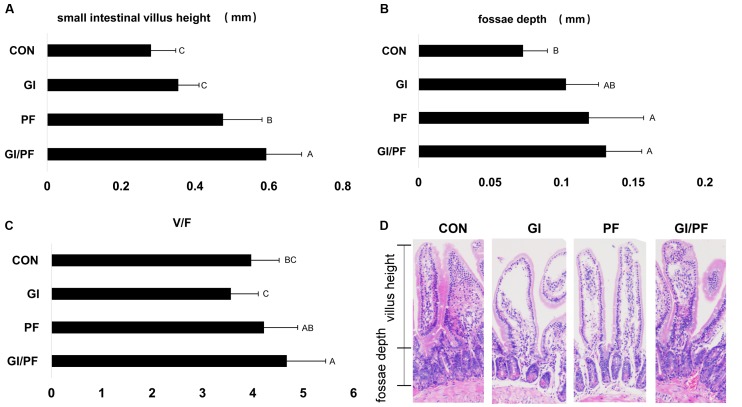
Effect of the three treatments on small intestinal villus height and fossae depth at 4-week point (mm). **(A)** Small intestinal villus height (mm). **(B)** Fossae depth (mm). **(C)** The ratio of the small intestinal villus height and fossae depth. **(D)** Photomicrographs of small intestinal villus structure. The data represent the mean ± SD. Letters A–C denote significant differences (*p* < 0.05) among different treatments. Selecting 10 villus height and fossae depth for measurement.

Gut bacteria may affect intestinal villus morphology as well as causing changes at the cellular level. The ratio of villus height to crypt depth (V/F) reflects the functional status of the small intestine, with a decreased ratio indicating mucosal damage, which results in impaired digestion and absorption. After 3 weeks of supplementation, the V/F values of the PF and GI/PF mice were significantly higher than those of the GI and control mice (*p* < 0.05, **Figure 5**).

## Discussion

GOS and inulin are effective prebiotics because they resist digestion in the small intestine but are fermented within the colon. They play important roles in adjusting the composition of the intestinal microbiota, maintaining the normal intestinal environment, regulating intestinal function, and improving overall human health (Kerperien et al., 2014). Polyglucose is a water-soluble dietary fiber that can reduce the emptying time of the stomach, effectively improving intestinal function. Polyglucose is also only fermented in the lower part of the gastrointestinal tract, producing SCFA and decreasing the intestinal pH (Vrese and Schrezenmeir, 2008). The insoluble dietary fiber extracted from bran cannot dissolve in water and is therefore not hydrolyzed by microorganisms in the large intestine. However, it does reduce the residence time of the excrement in the intestine by increasing the volume of the feces, and thereby acts as a laxative. In this study, we examined the effects of GI, PF, and GI/PF treatment on mouse intestinal microflora composition, fecal metabolic phenotype, cecal content pH, the V/F ratio in the small intestine, and the concentrations of SCFA, DAO, and TMAO in serum.

After 3 weeks of GI supplementation, we found that the proportion of *Bacteroides* species was significantly increased, while *Alloprevotella* were significantly decreased. *Bifidobacterium* species were also significantly increased, but the proportion was very small. In contrast, PF supplementation significantly reduced the proportion of *Bacteroides* but increased the numbers of *Alloprevotella* and *Parabacteroides*. Interestingly, we observed fluctuations in the dominant genus (*Bacteroides*, *Alloprevotella*) of GI/PF-fed animals over the experimental period, although the changes were not significant (*p* > 0.05). Together, these findings indicate that the different dietary supplements could significantly affect the composition of the intestinal microbiota. Functional oligosaccharide and/or ordinary dietary fiber intervention also changed the fecal metabolic profile. Changes in the intestinal microbiota associated with metabolic changes have been used to understand the possible mechanisms of health and disease progression (Arrieta et al., 2015; Shi et al., 2015; Del Chierico et al., 2016). The fecal metabolites of the GI/PF group varied in the range of small fluctuations, which may be closely related to the diversity of the intestinal microbiota. However, the observed changes in the fecal metabolites of the GI and PF groups were large. Decreasing diversity leads to significant changes in fecal metabolites, which is detrimental to health.

And 5–10 years before the onset of diabetes, some of the amino acid content is increased, especially isoleucine, leucine, valine, tyrosine, and phenylalanine (Wang T.J. et al., 2011). Our animal experiments clearly showed that these five amino acids are significantly reduced with the rational combination of components. It is important to control and reduce the incidence of diabetes. For the prediction of the risk of diabetes and heart disease, assessment of these amino acids plays a good preventive effect.

The pH of the colon contents was significantly decreased (*p* < 0.05) in all three groups over the experimental period, and the GI and GI/PF groups also showed significant increases in concentrations of SCFA in the colon contents. This may be the result of oligosaccharide fermentation producing a large number of SCFA, which would decrease the pH. The SCFA concentration in the colon contents of the GI/PF group did not change significantly, but this was not significantly related to the fluctuations in the dominant bacteria in this group. The increase in DAO concentration in the serum of GI-fed mice indicated an increase in intestinal permeability, as previous work has shown that increased levels of oligosaccharides increase the intestinal permeability of mice (Minten et al., 2009). The concentration of DAO in the serum of the PF group did not change significantly during the intervention period, but levels significantly decreased upon completion of the 3-week GI/PF feeding period (*p* < 0.05). Metabolite TMAO plays an important role in the development of cardiovascular disease, in which elevated TMAO levels are positively correlated with cardiovascular disease. Intestinal microbes produce trimethylamine lyase, an enzyme not produced by mammals, which cleaves the C–N bond of TMAO, with the resulting “metabolic waste” released through the portal vein into the liver (Wang Z. et al., 2011). In the liver, the cleaved TMAO is oxidized by flavin monooxygenase (Kaysen et al., 2015; Romano et al., 2015). The levels of TMAO in the serum were significantly increased in mice after 3 weeks of supplementation with GI or PF (*p* < 0.05), while no significant change was observed in the GI/PF group. This suggests that supplementation with a single type of polysaccharides can cause dramatic changes in the intestinal microbiota of mice, resulting in damage to the intestinal microecological balance, increased intestinal permeability, and increased TMAO content.

The small intestine is the main organ involved in nutrient digestion, absorption, and transport, so a healthy mucosal structure is particularly important for digestive physiological function, and overall growth and development of the body. The villi are an important part of the small intestine, where they are essential for the absorption of nutrients (Caspary, 1992). However, colonization of harmful bacteria can cause damage to the intestinal villus structure. The intestinal microbiota may also cause changes at the cellular level, in addition to alterations in intestinal villus morphology (Thoreux et al., 1998). The V/F ratio reflects the functional status of the small intestine: a decrease in the ratio indicates that the mucosa is damaged, decreasing rates of digestion and absorption, and ultimately obstructing growth and development of the animal. After 3 weeks of GI/PF supplementation, the V/F ratio was significantly higher than that of the control and GI group. While the ratio was not significantly altered compared with the control in the GI group, there was a slight decrease. This may be the result of changes in the bacterial composition caused by oligosaccharides and ordinary dietary.

## Conclusion

Maintaining the diversity of intestinal microbiota and abundance of dominant bacteria is extremely important for the health and supplementation with a single type of polysaccharides may cause dramatic changes in the intestinal microbiota, resulting in damage to the intestinal microecological balance. The addition of a combination of oligosaccharides and dietary fiber helps maintain the stability of the intestinal microecosystem.

## Author Contributions

WC and AJ designed research; WC, JL, BL, and WL performed research; WC, XW, JY, MC, and ZZ contributed new reagents or analytic tools; WC, CS, WB, and BY analyzed data; WC and JL wrote the paper.

## Conflict of Interest Statement

The authors declare that the research was conducted in the absence of any commercial or financial relationships that could be construed as a potential conflict of interest.

## Footnotes

1 http://drive5.com/uparse/

2 http://rdp.cme.msu.edu/
