# Supplementary material for: Effect of Functional Oligosaccharides and Ordinary Dietary Fiber on Intestinal Microbiota Diversity
Source: Front Microbiol. 2017 Sep 20;8:1750. doi: 10.3389/fmicb.2017.01750 (PMC5611707; doi:10.3389/fmicb.2017.01750)
Supplement: Supplementary file 1 [file Table_1.DOCX]

**Table S1.** Characteristics of the 16 metabolites identified differentially between experimental groups and the control.

| Metabolites | GI1/CON | |  | PF1/CON | |  | GIPF1/CON | |  | GI2/CON | |  | PF2/CON | |  | GIPF2/CON | |
| --- | --- | --- | --- | --- | --- | --- | --- | --- | --- | --- | --- | --- | --- | --- | --- | --- | --- |
|  | VIP | P-VALUE |  | VIP | P-VALUE |  | VIP | P-VALUE |  | VIP | P-VALUE |  | VIP | P-VALUE |  | VIP | P-VALUE |
| Succinic Acid | 1.441 | 0.02 |  | 1.998 | 0.007 |  | 1.192 | 0.038 |  | 1.513 | 0.004 |  | 1.007 | 0.003 |  | 1.257 | 0.003 |
| Proline | 1.869 | 0.001 |  | 1.033 | 0.003 |  | 1.83 | 0.019 |  | 1.815 | 0.002 |  | 1.749 | 0.019 |  | 1.282 | 0.03 |
| 9,12-Octadecadienoic acid | 1.734 | 0.009 |  | 1.704 | 0.013 |  | 1.504 | 0.019 |  | 1.571 | 0.035 |  | 1.024 | 0.005 |  | 1.034 | 0.026 |
| Myo-Inositol | 1.435 | 0.005 |  | 1.295 | 0.017 |  | 1.03 | 0.006 |  | 1.234 | 0.015 |  | 1.155 | 0.041 |  | 1.71 | 0.001 |
| Cholesterol | 1.81 | 0.013 |  | 1.367 | 0.007 |  | 1.836 | 0.008 |  | 2.072 | 0.01 |  | 1.746 | 0.034 |  | 1.539 | 0.035 |
| α-Hydroxyglutaric acid | 1.249 | 0.001 |  | 1.499 | 0.035 |  | 1.456 | 0.003 |  | 1.604 | 0.019 |  | 1.746 | 0.004 |  | 1.028 | 0.022 |
| Glycine | 1.389 | 0.017 |  | 1.501 | 0.034 |  | 1.491 | 0.025 |  | 1.019 | 0.013 |  | 1.773 | 0.002 |  | 1.58 | 0.012 |
| 2-Hydroxybutanoic acid | 1.796 | 0.015 |  | 1.712 | 0.004 |  | 1.789 | 0.001 |  | 1.786 | 0.001 |  | 1.69 | 0.014 |  | 1.124 | 0.018 |
| Glucose | 1.109 | 0.033 |  | 1.763 | 0.012 |  | 1.777 | 0.014 |  | 1.629 | 0.037 |  | 1.772 | 0.002 |  | 1.072 | 0.007 |
| Pyruvic acid |  |  |  |  |  |  |  |  |  | 1.161 | 0.027 |  | 1.128 | 0.041 |  | 1.668 | 0.006 |
| Lactic acid | 1.538 | 0.01 |  | 1.764 | 0.023 |  | 2.065 | 0.032 |  | 1.63 | 0.005 |  | 1.973 | 0.032 |  | 1.833 | 0.002 |
| Octadecanoic acid | 1.141 | 0.011 |  | 1.177 | 0.008 |  | 1.882 | 0.002 |  | 1.786 | 0.001 |  | 1.767 | 0.017 |  | 1.743 | 0.007 |
| L-Aspartic acid | 1.257 | 0.024 |  | 1.095 | 0.012 |  | 1.482 | 0.03 |  | 1.786 | 0.001 |  | 1.197 | 0.023 |  | 1.656 | 0.039 |
| 5-Oxoproline | 1.046 | 0.017 |  | 1.524 | 0.048 |  | 1.957 | 0.003 |  | 1.4 | 0.016 |  | 1.069 | 0.001 |  | 1.529 | 0.037 |
| Mannose | 1.412 | 0.003 |  | 1.002 | 0.039 |  | 1.644 | 0 |  | 1.786 | 0.001 |  | 1.794 | 0.003 |  | 1.091 | 0 |
| Galactose | 1.041 | 0.002 |  | 1.524 | 0.037 |  | 1.514 | 0.009 |  | 2.054 | 0.029 |  | 1.057 | 0.013 |  | 1.753 | 0.005 |
