# Supplementary material for: Effect of Functional Oligosaccharides and Ordinary Dietary Fiber on Intestinal Microbiota Diversity
Source: Front Microbiol. 2017 Sep 20;8:1750. doi: 10.3389/fmicb.2017.01750 (PMC5611707; doi:10.3389/fmicb.2017.01750)
Supplement: Supplementary file 2 [file Table_2.DOCX]

| Metabolites | GI1/CON | PF1/CON | GI+PF1/CON | GI2/CON | PF2/CON | GI+PF2/CON | pathways |
| --- | --- | --- | --- | --- | --- | --- | --- |
| L-Isoleucine | 0.21 | 0.15 | 0.16 | 0.46 | 0.56 | 0.28 | Valine, leucine and isoleucine degradation |
| L-Leucine | -- | 2.15 | 0.11 | 3.03 | 1.63 | 0.65 | Valine, leucine and isoleucine degradation |
| Norvaline | 1.44 | 1.01 | 0.66 | 2.08 | 1.22 | 0.60 | -- |
| L-Tyrosine | 0.35 | 0.37 | 0.30 | -- | -- | 0.78 | Phenylalanine and tyrosine metabolism |
| L-phenylalanine | 0.13 | 0.16 | 0.14 | 0.52 | 0.32 | 0.18 | Phenylalanine and tyrosine metabolism |

**Table S2.** Trends in levels of Amino acids in experimental mice

GI1/CON: the ratio of the values from GI and Control animals after 2 weeks of supplementation, indicating fold change in the level of metabolites. PF1/CON: the

ratio of the values from PF and Control animals after 2 weeks of supplementation. GIPF1/CON: the ratio of the values from GIPF and Control animals after 2 weeks

of supplementation. GI2/CON: the ratio of the values from GI and Control animals after 3 weeks of supplementation, indicating fold change in the level of metabolites.PF2/CON: the ratio of the values from PF and Control animals after 3 weeks of supplementation.GIPF2/CON: the ratio of the values from GIPF and Control animals after 3 weeks of supplementation.
